# Amino acid transporter expansions associated with the evolution of obligate endosymbiosis in sap-feeding insects (Hemiptera: sternorrhyncha)

**DOI:** 10.1186/s12862-015-0315-3

**Published:** 2015-03-25

**Authors:** Romain A Dahan, Rebecca P Duncan, Alex CC Wilson, Liliana M Dávalos

**Affiliations:** Department of Ecology and Evolution, State University of New York at Stony Brook, Stony Brook, NY 11794 USA; Department of Biology, University of Rochester, Rochester, NY 14627 USA; Department of Biology, University of Miami, Coral Gables, FL 33146 USA; Consortium for Inter-Disciplinary Environmental Research (CIDER), State University of New York at Stony Brook, Stony Brook, NY 11794 USA

**Keywords:** Gene family evolution, Endosymbiosis, Gene duplication, Phylogenetics

## Abstract

**Background:**

Mutualistic obligate endosymbioses shape the evolution of endosymbiont genomes, but their impact on host genomes remains unclear. Insects of the sub-order Sternorrhyncha (Hemiptera) depend on bacterial endosymbionts for essential amino acids present at low abundances in their phloem-based diet. This obligate dependency has been proposed to explain why multiple amino acid transporter genes are maintained in the genomes of the insect hosts. We implemented phylogenetic comparative methods to test whether amino acid transporters have proliferated in sternorrhynchan genomes at rates grater than expected by chance.

**Results:**

By applying a series of methods to reconcile gene and species trees, inferring the size of gene families in ancestral lineages, and simulating the null process of birth and death in multi-gene families, we uncovered a 10-fold increase in duplication rate in the AAAP family of amino acid transporters within Sternorrhyncha. This gene family expansion was unmatched in other closely related clades lacking endosymbionts that provide essential amino acids.

**Conclusions:**

Our findings support the influence of obligate endosymbioses on host genome evolution by both inferring significant expansions of gene families involved in symbiotic interactions, and discovering increases in the rate of duplication associated with multiple emergences of obligate symbiosis in Sternorrhyncha.

**Electronic supplementary material:**

The online version of this article (doi:10.1186/s12862-015-0315-3) contains supplementary material, which is available to authorized users.

## Background

Nutritional mutualistic endosymbioses are characteristic of sap-feeding insections of the sub-order Sternorrhyncha (Hemiptera), including aphids, scales, whiteflies and psyllids [1], and the closely related Auchenorrhyncha that includes cicadas [2]. The obligate primary endosymbionts of sap-feeding insects provide their hosts with essential amino acids [3,4]. Nutritional symbioses are also found in blood-feeding insects such as the kissing bug, *Rhodnius prolixus*, and the human body louse, *Pediculus humanus.* These blood-feeders obtain vitamins from their bacterial symbionts [5-7]. Genomic evolution of symbionts toward reduced gene content, AT bias, and predictable gene sets based on the nutritional roles of symbionts has been repeatedly confirmed. In contrast, genomic signatures of symbiosis are only now being revealed in host genomes [8-11].

Transport of essential amino acids between symbionts and sternorrhynchan hosts at the symbiotic interface is mediated by amino acid transporters from two gene families: the amino acid polyamine organocation transporters (APC; Transporter Classification #2.A.3); and the amino acid/auxin permease transporters (AAAP; TC #2.A.18) [8,10]. Multiple genes identified in these families have been duplicated, and some paralogs are known to be expressed at the symbiotic interface in two sternorrhynchans: the pea aphid *Acyrthosiphon pisum*, and the citrus mealybug *Planococcus citri* [8,10]. The expression of duplicated amino acid transporters in the bacteriocytes (insect cells that house symbionts) of *A. pisum* and *P. citri* suggests duplication provided new genes, and thereby facilitated recruitment of amino acid transporters to operate in the novel context of the host/symbiont interface. If gene duplication in amino acid transporters is important for interactions between sternorrhynchan insects and their symbionts, then selection should favor the retention and subsequent recruitment of new paralogs for amino acid exchange at the symbiotic interface. In contrast, related blood-feeding species would not be expected to expand amino acid transporter families, as their nutritional constraints involve vitamins; and auchenorrhynchans may or may not expand their transporters, depending on the constraints they experienced in their independently evolved nutritional symbioses.

Duncan et al. [10] observed that amino acid transporter families appear to have undergone clade-specific expansions in Sternorrhyncha. They hypothesized these expansions resulted from selection for the maintenance of paralogs to mediate amino acid exchange at the host/symbiont interface. Their hypothesis was not formally tested [10], and comparative, quantitative analyses are necessary for accurate investigation of the evolution of host/symbiont coevolution in this sub-order. While the hypothesized clade-specific expansions in Sternorrhyncha could be attributed to the most recent common ancestor of extant stenorrhynchan insects, multiple lines of evidence support independent coevolution of host/symbiont genomes in the four main sternorrhynchan families [8-14]. Additionally, phylogenetic analyses strongly suggest that sap-feeding nutritional symbioses have evolved multiple times in Hemiptera, including independent origins in Sternorrhyncha (phloem sap-feeders) and Auchenorrhyncha (xylem or phloem sap-feeders) [2]. Therefore analyses of gene family evolution in this system are best interpreted as the result of multiple instances of selection in several independent lineages, as opposed to a small number of events traceable to a common ancestor. Here, we provide statistical tests of the hypothesis that expansions of amino acid transporter genes in sap-feeding sternorrhynchan resulted from selection for an increased number of paralogs in lineages that evolved nutritional endosymbiosis. To this end, we use comparative methods and introduce a new pipeline for applying a wide range of analyses of gene family evolution.

Inference of the evolution of amino acid transporters in sternorrhynchan insects requires a representative taxonomic sample. A complete, resolved insect phylogeny was absent from Duncan et al. [10], and is necessary to infer the evolutionary history of relevant gene families in insects with a variety of diets and lifestyles. A recent phylogenomic analysis has resolved the general topology and timing of insect evolution, including the Hemiptera [15]. That study, however, did not include all the sternorrhynchan taxa needed to test for amino acid transporter expansions. Here, as the basis for comparative analyses of the evolution of amino acid transporters in Sternorrhyncha, we infer a phylogeny of insects encompassing Paraneoptera and Holometabola. We include representatives from the four major sternorrhynchan families, as well as an auchenorrhynchan xylem sap-feeder, a heteropteran blood-feeder, and additional taxa to improve the statistical power of comparative methods.

Phylogenetic methods provide an array of powerful techniques to infer the evolutionary history of gene family evolution (for a detailed review of these methods, see [16]). Methods for reconciling gene trees to species phylogenies using parsimony to infer the history of gene duplications and losses are well-established [17-19]. Such methods, however, may be biased if the gene tree is not well resolved or supported [20]. Alternatively, non-reconciliation techniques infer the history of gene families from the number of genes found in extant species. Parsimony, maximum likelihood and Bayesian algorithms have been implemented to test for deviation from a null birth-death model of gene family evolution [21-25]. Here, we use both reconciliation and non-reconciliation techniques coupled with simulations of birth-death models of gene evolution [26,27] to analyze the history of two amino acid transporter gene families in phloem-feeding sternorrhynchans.

## Results

### Phylogenetic inference

**Figure 1:**
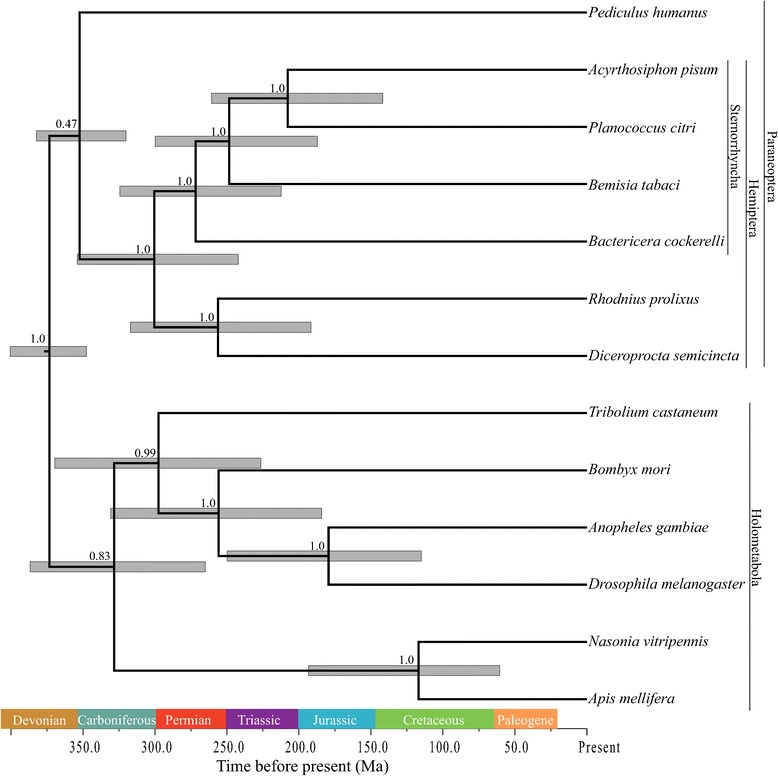
**Bayesian phylogeny inferred using 20 genes of 13 species**. The median posterior likelihood was-160204. Node bars represent the 95% confidence interval for the age of the nodes. The corresponding geological eras are given for reference, and colored according to the Commission for the Geological Map of the World (CGMW), Paris, France.

Both the maximum likelihood (ML) and Bayesian species phylogenies with 10 partitions recovered Sternorrhyncha as a monophyletic clade within Hemiptera (Table 1, Figure 1, Additional file 1: Figure S1, bootstrap support [bs] = 100, posterior probability [pp] = 0.9995). Auchenorrhyncha (represented by *Diceroprocta semicincta*) and Heteroptera (represented by *Rhodnius prolixus*) formed a clade sister to Sternorrhyncha (Figure 1, bs = 100, pp = 0.9955). Psocodea (represented by *Pediculus humanus*) was sister to Hemiptera in the Bayesian phylogeny, making Paraneoptera monophyletic, although with low support (pp = 0.47, Figure 1). This result was obtained despite the starting ML phylogeny including a paraphyletic Paraneoptera as in [15] (Additional file 1: Figure S2).

**Table 1:** **Genes used in the phylogenetic reconstruction, with accession numbers from the OrthoDB database and inferred substitution matrices**

**Partition**	**OrthoDB accession**	**Gene/protein**	**Substitution Matrix**
1	EOG7W4CH6	Transcription factor 2S	Le-Gascuel (LG) [54]
	EOG7JQQ2Q	Uncharacterized protein	
	EOG72S0DN	GRIM-19	
2	EOG7B94KB	Mago-Nashi	Jones-Taylor-Thornton (JTT) [55]
	EOG7P0F0G	Translation Initiation Factor 5A	
3	EOG771DPV	Ribosomal Protein L30	LG
	EOG73C6VD	60S Ribosomal Protein L31	
	EOG74FSG2	Ubiquitin-conjugating enzyme	
	EOG73JZB1	Ribosomal Protein S16	
4	EOG7W1HKQ	Ribosomal Protein S26	Müller-Vingron (VT) [56]
5	EOG7VTRRV	tRNA Synthetase	LG
	EOG7ND679	Exonuclease, RNAse T/DNA Polymerase III	
6	EOG74RCV4	Cleft lip and palate Transmembrane I	LG
	EOG7748KP	Succinyl-CoA: 3-Ketoacid-coenzyme A transferase	
	EOG7Z9HRH	Uncharacterized protein	
7	EOG73ZDZ5	Gtr1/Rag AG protein	JTT
	EOG7SFW14	Uncharacterized protein	
8	EOG7455DF	Chloride Channel	LG
9	EOG7P38WC	Tetratricopeptide repeat	VT
10	EOG799DV6	Pyridoxal-phosphate-dependent Transferase	LG

Alignment and phylogenetic data are available on TreeBase.

### Evolution of amino acid transporter gene families

**Figure 2:**
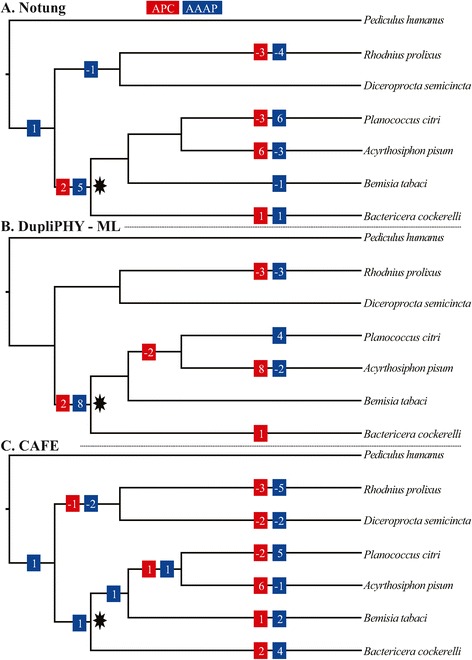
**Expansions of amino acid transporters families in the chronogram of Hemiptera**. Expansions and contractions in APC and AAAP inferred using **A:** Notung [18,19]; **B:** DupliPHY-ML [22]; and **C:** CAFE [24,25]. The star represents the most recent common ancestor (MRCA) of Sternorrhyncha. Expansions and contractions outside of Hemiptera were omitted for clarity.

**Figure 3:**
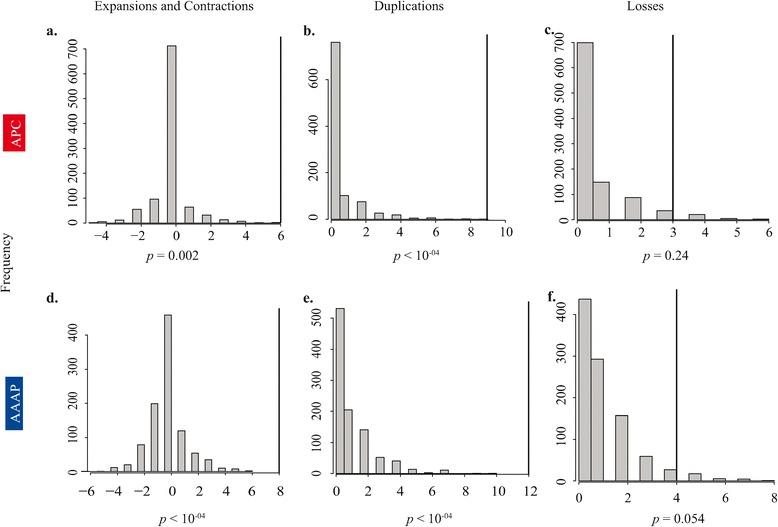
**Simulations of gene family evolution in Hemiptera.** Null distributions of net expansion or contraction, duplications, and losses of two amino acid transporter gene families within Sternorrhyncha from 1000 simulations of a birth-death model of evolution using GenPhyloData [27]. Expansion or contraction was obtained by subtracting the number of losses from the number of duplications detected in Sternorrhyncha in a single replicate. **A**. Expansion or contraction in APC and **D**: AAAP in; **B**. duplications in APC and **E**: in AAAP; **C**. losses in APC and **F**: in AAAP. The thick black lines represent the values inferred using Notung.

Relative to other insects, sternorrhynchans had more amino acid transporter paralogs (Figure 2, Table 2). Both the type of analysis and optimization influenced estimates of the history of amino acid transporter gene families. Notung (reconciliation and parsimony) and DupliPhy-ML (gene copy number and ML) inferred large family expansions or contractions toward the tips of the species phylogeny, as well as a large expansion of the AAAP family at the most recent common ancestor (MRCA) of Sternorrhyncha (Figure 2). In contrast, CAFE (gene copy number and ML) favored a model inferring many smaller-scale expansions throughout the clade (Figure 2). Despite analytical differences, all approaches inferred expansions (a net increase in the size of the gene family, obtained by subtracting the number of losses from the number of duplications in the clade, noted k subsequently) of both amino acid transporter gene families in Sternorrhyncha beyond what is expected from the null birth-death model (Figure 3, APC [net expansion inferred in all branches of the clade] k = 6, *p* = 0.002; AAAP k = 8, *p* < 10^-4^). Subsequent simulations showed these expansions were the result of more duplications —as opposed to fewer losses— relative to the expectations of a simulated birth-death model (Figure 3). Notung provided the most conservative estimates of the number of duplications and losses in Sternorrhyncha, and we used its results to compare against the simulated distributions (Figure 2).

**Table 2:** **Taxa set for phylogenetic analyses and number of gene copies for both APC and AAAP transporter superfamilies for each species**

**Species**	**Common name**	**APC loci**	**AAAP loci**
*Acyrthosiphon pisum*	pea aphid	18	22
*Planococcus citri*	citrus mealybug	10	28
*Bemisia tabaci*	whitefly	12	24
*Bactericera cockerelli*	potato psyllid	13	25
*Diceroprocta semicincta*	Cicada sp.	10	16
*Rhodnius prolixus*	kissing bug	7	13
*Pediculus humanus*	human body louse	8	16
*Tribolium castaneum*	red flour beetle	10	16
*Nasonia vitripennis*	jewel wasp	10	12
*Apis mellifera*	honeybee	14	14
*Bombyx mori*	silk moth	12	16
*Anopheles gambiae*	mosquito	9	15
*Drosophila melanogaster*	fruit fly	10	17

(Data from Price et al. (2011) [8] and Duncan et al. (2014) [10]).

The model-fitting approach implemented in CAFE allowed us to compare models with a single birth-death parameter λ throughout the phylogeny against models with the λ rate shifting in certain clades using likelihood-ratio tests. A model in which λ was allowed to shift in Sternorrhyncha was favored over the null model with a single λ for the AAAP family (Likelihood Ratio [LR] = 6.63, *p* = 0.008), but not in the APC family (LR = 1.56, *p* = 0.204; Table 3). Under the better-fit model, the duplication rate in Sternorrhyncha increased ~11X above the background rate of the phylogeny for the AAAP family (Table 3). A structured model allowing for different rates in Sternorrhyncha and differentiating between the rate of duplication λ and the rate of gene death μ was the best fit for the AAAP family (LR = 8.46, *p* = 0.002, Table 4). This more complex model inferred an increase in λ over 8 orders of magnitude for AAAP in Sternorrhyncha, while the rate of gene death μ decreased to 0.003 of the background rate (Table 4). In APC, a model differentiating between a global λ and a global μ across the phylogeny did not explain the data significantly better than the simpler null model (LR = 0.06, *p* = 0.843; Table 4). The results of models using an alternate tree topology with *P. humanus* more closely related to Holometabola, as inferred in [15,28], were highly comparable, resulting in the same models being selected in all cases (see Additional file 1: Table S1 and S2).

**Table 3:** **Results of likelihood-ratio tests comparing null models to those allowing a rate shift in the branches in Sternorrhyncha**

	**Model**	**λ**_**Background**_ **x 10** ^**3**^	**λ**_**Sternorrhyncha**_ **x 10** ^**3**^	**Fold increase**	**-ML**	**# Parameters**	**Likelihood ratio**	***p-*** **value**
APC	Single λ	1.2177	-	-	34.31	1	1.562	0.204
	Multiple λ	0.7307	2.5049	3.43	33.52	2		
AAAP	Single λ	1.3119	-	-	38.33	1	6.633	0.008
	Multiple λ	0.3286	3.6802	11.20	35.01	2		

λ represents the probability of gene duplication and loss per gene per million years.

**Table 4:** **Results of the Likelihood-ratio test comparing models differentiating** λ **and** μ **to models with equal birth-death parameters**

	**Model**	**λ**_**Background**_ **x 10** ^**3**^	**λ**_**Sternorrhyncha**_ **x 10** ^**3**^	**μ**_**Background**_ **x 10** ^**3**^	**μ**_**Sternorrhyncha**_ **x 10** ^**3**^	**-ML**	**# Parameters**	**Likelihood ratio**	***p*** **-value**
APC	Single λ	1.2177	-	-	-	34.31	1	0.06	0.843
	Single λ + μ	1.2533	-	1.0615	-	34.28	2		
AAAP	Multiple λ	0.3631	6.849	-	-	35.01	2	12.51	0.002
	Multiple λ + μ	2.88 x 10^-9^	1.110	0.6341	6.03 x 10^-3^	28.76	4		

For APC, the estimated parameters are global across the phylogeny. For AAAP, the parameters were rates were allowed to shift in sternorrhynchan species. Data generated with CAFE. λ represents the probability of gene duplication/loss (Single/Multiple λ), or gene duplication only (Single/Multiple λ + μ) per gene per million years. μ represents the probability of gene loss per gene per million years.

## Discussion

We implemented phylogenetic comparative methods to infer the evolutionary history of amino acid transporter gene families, and test their association with the evolution of nutritional symbioses in Sternorrhyncha. Using a range of approaches, we found Sternorrhyncha-specific expansions of both the APC and AAAP amino acid transporter families. Notably, within the AAAP family the Sternorrhyncha-specific expansions were associated with a steep increase in the duplication rate and decrease in the rate of gene loss. These results provide strong support for our hypothesis that expansions of amino acid transporters were favored in sap-feeding sternorrhynchan lineages relying on endosymbiosis for essential amino acid provisioning. Crucially, the results are robust to both genome annotation and phylogenetic uncertainty, as summarized below.

### Genome annotation and phylogenetic uncertainty

Our analyses are robust despite the potential for uneven sampling of amino acid transporters across lineages. The method used by Duncan et al. [10] to identify transporters was highly conservative, and we are confident that closely related genes represent true paralogs, and not allele or splice variants. For example, all transcripts from the citrus mealybug *P. citri* were mapped to its draft genome, and any transcripts sharing at least one exon were collapsed into a single representative locus. For the remaining hemipterans in which duplications were inferred (*B. cockerelli*, *Be. tabaci*), Duncan et al. [10] used the Goldman and Yang method [29] to calculate the pairwise rate of synonymous substitutions (*dS*) between closely related genes within a species and collapsed transcript sets with pairwise *dS* of 0.25 or less. This cutoff value is equivalent to the pairwise *dS* of orthologs between two aphids, *A. pisum* and *Myzus persicae*, and represents a divergence of 32 to 53 million years [30,31]. The *dS* ≤ 0.25 cutoff provides a highly conservative estimate for the number of amino acid transporter loci. For example, three recently duplicated true paralogs in the APC family of amino acid transporters collapsed into one representative locus in *A. pisum* [10]. Sampled hemipterans could have more true amino acid transporter paralogs than estimated, but not fewer. This underestimation of the number of amino acid transporter paralogs will reduce the signals of expansion in analyses, highlighting the robustness of our results. Finally, our findings are robust despite uncertainties pertaining to the general topology of the taxonomic groups used here, in particular the placement of Psocodea relative to Paraneoptera and Holometabola [15,28,32].

### Amino acid transporter expansions

We detected a significant expansion of the APC family of amino acid transporters in Sternorrhyncha, inconsistent with a neutral birth-death model of gene family evolution. Simulations-based tests revealed that there were more gene duplication events in the clade than expected, but no more or fewer gene losses. However, the best-fit model of evolution inferred by CAFE was a null model in which a single rate of gene duplication/death governed the APC family across the phylogeny. This apparent discrepancy in the results may be explained in one of several ways: (1): Since the changes are happening in a relatively small clade in the phylogeny the inference method implemented in CAFE may not be powerful enough to detect a finer-scale change that may be occurring; or (2): Gene family evolution through duplication and loss is an inherently stochastic process, and the simulations may have detected random variation in the APC family size as a result of the low variance in non-stenorrhynchan taxa in the phylogeny. In general, phylogenetic model comparisons based on simulations (e.g., [33]) tend to be more sensitive than comparisons based on model fit (e.g., [34]). If this were the case, a larger sample of insect species is needed to directly estimate differential rates of birth and death along branches in the phylogeny using CAFE.

In contrast, in the AAAP family, rates inferred using CAFE and the results of simulations were consistent. We detected a net significant expansion —more duplications than losses— in the family in Sternorrhyncha compared to expected values (Figure 3). CAFE’s best-fit model for AAAP was one in which the rate of gene duplication vastly exceeded the rate of gene death, with both rates shifting in Sternorrhyncha (Table 4). These results are inconsistent with neutral gene family evolution.

Under a neutral or nearly neutral process, paralogs arising from gene duplications are expected to experience relaxed selection, eventually leading to subfunctionalization and pseudogenization or (rarely) neofunctionalization. This is the stochastic birth-death model of gene family evolution [26,35]. Rejecting a stochastic birth-death model implies that a non-neutral process governs the evolutionary fate of duplicate genes on a given branch of a phylogeny. Our results for the AAAP family of amino acid transporters support the hypothesis that duplicate genes provide a selective advantage to sternorrhynchan lineages, but not to other insects. Despite differences in the mode of evolution inferred, this result was consistent using three different methods to infer the history of amino acid transporter gene families in our phylogeny thus, revealing a strong underlying signal. Therefore, Sternorrhynchan insects appear to have been strongly selected for an increased number of AAAP family amino acid transporters. The simulations support a similar explanation for the persistence of APC paralogs in sternorrhynchans, although the mode of evolution of the APC family remains unknown.

Recent evidence suggests that amino acid transporter family expansions would benefit sternorrhynchans by mediating the transport of amino acids between hosts and symbionts [8,10,36]. Since sternorrhynchan endosymbiotic bacteria have few, if any, amino acid transporters in their genomes [37,38], transport at the symbiotic interface must operate via host genome encoded amino acid transporters. Transcriptomic analyses show that sternorrhynchans with amino acid-provisioning symbionts have amino acid transporter paralogs expressed at their symbiotic interface, supporting a role for host paralogs in nutritional symbiosis [10]. These observations, combined with formal statistical tests for deviation from a neutral birth-death model, support the hypothesis that selection maintains amino acid transporter paralogs in sternorrhynchan insections By demonstrating that amino acid transporter gene families underwent expansions in this sub-order, our results provide further evidence for the selective maintenance of amino acid transporter paralogs of the AAAP, and perhaps APC, gene families in sternorrhynchan insections We propose that selection arises from evolutionary constraints for novel transporters with specialized roles in mediating symbiotic amino acid exchange.

### Evolution of endosymbiosis

A range of genomic, transcriptomic, and comparative data suggest that amino acid transporters have been retained because species have evolved endosymbiotic mutualisms and experienced concomitant expansions in amino acid transporter gene families, and not because the MRCA of Sternorrhyncha evolved an endosymbiotic, sap-feeding lifestyle. The monophyly of Sternorrhyncha is strongly supported, both in our phylogenetic inference and in the literature [15,39,40]. Based on shared characteristics of sternorrhynchans, previous studies of amino acid transporters have sometimes assumed that extant Sternorrhyncha shared a symbiotic, phloem-feeding ancestor [8,10], but this interpretation is inconsistent with current data. For example, psyllids and mealybugs have experienced independent horizontal gene transfers of bacterial genes involved in essential amino acid synthesis [9,11]. Additionally, variations in host/symbiont metabolic complementarity in different sternorrhynchan lineages support the hypothesis of multiple origins for nutritional symbiosis. For example, aphid and mealybug bacterial symbionts are both missing the *ilvE* gene, responsible for the terminal step in the biosynthesis of branch-chain amino acids. The final step in the biosynthesis is carried out by the host-encoded branch-chain amino acid transaminase in aphids [14], and the ortholog of this transaminase is enriched in the bacteriocytes of mealybugs, suggesting the same complementarity in both lineages [9,38]. In contrast, *ilvE* is present in the psyllid symbiont *Carsonella* and expression of the psyllid branch-chain amino acid transaminase is not enriched in psyllid bacteriocytes [11]. Finally, amino acid transporters expressed at the symbiotic interface in sampled sternorrhynchan species are not orthologous [10], implying that the expansions inferred are the result of independent, parallel evolution. Given the parallel evolution of similar symbiosis-related genomic and metabolic patterns in different sternorrhynchan superfamilies, coevolution between these insects and their symbionts appears to be dynamic and independent.

The distribution of multigene family expansions on the phylogeny depends strongly on the method used to infer evolution, and has the potential to complement gene expression analyses that suggest parallel evolution of amino acid transporter recruitment to the symbiotic interface. The mode of amino acid transporter family expansions inferred using CAFE, in which expansions accumulate throughout all branches of the clade, is consistent with the paralogy of the amino acid transporters recruited at the insect/symbiont interface in Sternorrhyncha. In contrast, reconciliation methods, and in particular those based on parsimony, tend to minimize the number of gene gains and losses. In unresolved trees, such as the ones analyzed here, this may infer more duplications towards the root, and therefore more losses towards the tips [20]. Because of this known bias, non-reconciliation methods may be better guides to the history of amino acid transporters in light of the independent evolution of symbiosis in different lineages. Following the model of evolution inferred using CAFE, expansions in the AAAP family correspond to the branches on the phylogeny in which each family evolved endosymbiosis, in line with the multiple-origins hypothesis. Additional expansions of amino acid transporters detected in *A. pisum* and *P. citri* that are independent of the expansion inferred in the MRCA of Sternorrhyncha are also consistent with the parallel evolution of host/symbiont metabolic integration across the clade.

Expanded sampling of amino acid transporters to include species with varying interdependence on symbionts will help uncover the mechanisms of amino acid transporter expansions in Sternorrhyncha —particularly for the APC gene family—, and improve our understanding of the mode of evolution of endosymbiosis. If, as we hypothesize, amino acid transporter expansions happen in tandem with the evolution of a primary nutritional endosymbiosis, then significant increases in paralogs will only be found in lineages that display such symbioses. Conversely, if expansions of amino acid transporters are still inferred at the Sternorrhynchan MRCA and are present in species that lack bacteriocyte-associated symbionts such as the grape phylloxera *Daktulosphaira vitifoliae* (Fitch), selection for the maintenance of amino acid transporter paralogs would be independent of endosymbiosis in Sternorrhycha. In the latter case, duplicate amino acid transporters would be retained in sternorrhynchans because of an unidentified requirement common to all sternorrhynchans and independent of the symbiotic lifestyle of the insect species.

## Conclusions

Detailed comparative analyses support the hypothesis that the expansion of amino acid transporters in Sternorrhyncha has been beneficial in the context of obligate mutualistic endosymbiosis. This highlights the interdependency and complementarity of genomes associated through obligate symbiosis. As bacterial symbionts tend toward reduced genomes, host genomes may change drastically in structure and composition to complement the elements lost in the symbiont and to support a novel, beneficial symbiotic relationship.

The combination of statistical approaches we used, including model fitting and a novel pipeline involving simulations under a stochastic birth-death process, can be readily deployed in future analyses of gene family evolution to test for non-neutrality. With more extensive taxon sampling, these methods can further elucidate the patterns of amino acid transporter evolution in symbiotic insects. Finally, our analyses can be applied to investigate genomic evolution in other symbiotic clades, such as Auchenorrhyncha and various blood-feeding insects.

## Methods

### Taxonomic sampling

To model the evolution of amino acid transporters, we estimated the phylogeny of 13 species in two super-orders of the class Insecta (Table 2) [8,10]. Analyses included species representative of the four major superfamilies in Sternorrhyncha: *Acyrthosiphon pisum* from the Aphidoidea*, Planococcus citri* from the Coccoidea*, Bemisia tabaci* from the Aleyrodoidea and *Bactericera cockerelli* from the Psylloidea*.* Sternorrhyncha outgroup hemipterans included the auchenorrhynchan *Diceroprocta semicincta* and the blood-feeding heteropteran *Rhodnius prolixus.* The blood feeder *Pediculus humanus* was included as the outgroup to Hemiptera in the super-order Paraneoptera. Members of Holometabola were included as a monophyletic outgroup that includes *Apis mellifera* and *Nasonia vitripennis*, *Tribolium castaneum*, *Bombyx mori*, *Drosophila melanogaster* and *Anopheles gambiae* (see Additional file 1: Table S1 for taxonomic details).

### Sequence compilation

Orthologous amino acid sequences for *A. pisum, R. prolixus, P. humanus, N. vitripennis, T. castaneum, B. mori, D. melanogaster* and *An. gambiae* were obtained from OrthoDB, an online database cataloguing orthologous genes for many taxa [41]. *Ap. mellifera* sequences were extracted from the latest genome assembly (4.5) on BeeBase [42,43]. Sequences from the other species were obtained by applying tBLASTn to the transcriptome of each remaining species [10,44,45], using previously identified orthologs as queries (BLAST databases size ranged from 39,280 sequences to 182,687 sequences, cut-off e-value was 10^-04^) [46]. Groups were discarded if they failed to produce hits below the cut-off e-value, or if they returned more than one sequence below the cut-off e-value. This produced a pool of 48 putatively orthologous genes, from which 20 were randomly selected for subsequent phylogenetic inference to reduce computational time (Table 1).

### Alignment and phylogenetic inference

Amino acid sequences in each group were aligned using MAFFT 7.045b under default settings [47]. Each alignment was visually inspected, and one alignment with a pairwise identity below 40% was discarded. Another group was then drawn randomly from the pool to replace the rejected gene. The alignments were concatenated and analyzed using PartitionFinderProtein to estimate the best-fit partition scheme and substitution models for the amino acid supermatrix [48]. A Maximum Likelihood (ML) phylogeny was inferred using RAxML (Randomized Axelerated ML) v. 7.2.6 with 200 bootstrap pseudoreplicates and using the best fit models of protein evolution and partitions (Table 1) [49].

The resulting ML tree was dated using *chronopl* in the R package ‘ape’ v. 3.0–11 [50], with calibration nodes shown in Additional file 1: Figure S2. The Yule pure-birth model of speciation was used as prior on the branching patterns and a lognormal uncorrelated relaxed clock model as prior on branch lengths in a Bayesian phylogenetic analysis using BEAST v. 1.7.5 [51], in the Cipres science gateway [52]. The dated phylogeny was used as the starting point in this inference (Additional file 1: Figure S1). Bayesian phylogenetic analyses ran Markov-Chain Monte-Carlo (MCMC) searches over one billion generations sampling every 1000 generations. Results shown are from 5 independent 20-million-generations runs with a burn-in of 2,000,000 (2000 trees) each.

### Inferring amino acid transporter duplications

The numbers of amino acid transporter paralogs in each sampled species were obtained from [8,10]. Three different approaches were used to infer the history of amino acid transporters in our phylogeny: (1) Notung v. 2.6 [18,19] was used to reconcile the gene trees of APC and AAAP families (obtained from Duncan et al. [10]) with the species phylogeny, assigning default values for costs of duplications (1.5) and losses (1.0); (2) DupliPhy-ML [22] was run online with default parameters, and the best models were selected using the Akaike Information Criterion (AIC, Additional file 1: Table S3); and (3) CAFE v. 3.1 [24,25] was used to infer the evolutionary histories of both gene families and estimate the values of corresponding λ birth-death and μ parameters.

A null model of duplication was implemented using GenPhyloData, a tool that simulates random “guest” trees along a known host phylogeny [27]. The estimates from the single-λ CAFE models were assigned to the birth and death parameters in simulations. This process was automated over 1000 replicates. The resulting null distributions of duplications, losses and net expansions/contractions of amino acid transporter families at each node were compared to the values inferred by Notung, as it yielded the most conservative estimates of expansions/contractions, duplications and losses in Sternorrhyncha. A significance level of α = 0.05 was applied. Simulations were implemented in UNIX using GenPhyloData, and data analyses were performed in R v. 3.1.0, using the package ‘ape’ [50,53] (scripts available in the Additional files 2 and 3). This new pipeline provides a powerful basis for investigating gene families that are expected to evolve under a non-neutral process.

### Duplication rates for amino acid transporters in Sternorrhyncha

CAFE allows a structured inference of the λ birth-death parameter in the host phylogeny, so that it is possible to compare a single-parameter model against more complex models with clades having different rates of birth and deaths. We inferred the APC and AAAP gene family evolution using CAFE by fitting null models with a single parameter throughout the host phylogeny, and comparing those to models in which λ was allowed to differ in branches past the Sternorrhyncha MRCA (Table 3). The models were then compared using likelihood-ratio tests by generating simulated null likelihood ratio distributions within CAFE. For the APC family, we compared the ‘global’ model to a model in which λ and the rate of gene death μ were allowed to differ from one another (as opposed to the simpler model where λ = μ), globally. For AAAP, we compared the best-fit multiple-λ model to one in which λ and μ were allowed to differ from one another, and where the rates were allowed to shift within Sternorrhyncha, using a likelihood-ratio test, and approximating the likelihood ratio distribution to a *χ*^2^ distribution with 2 degrees of freedom. These tests were repeated using an alternate topology of the underlying species phylogeny reflected in the calibrated ML phylogeny (Additional file 1: Figure S1).

### Availability of supporting data

The data supporting this article is available in the TreeBASE repository, accession number S17122 http://purl.org/phylo/treebase/phylows/study/TB2:S17122

## Additional files

Additional file 1:
**Supplementary methods and displays.**
Additional file 2:
**UNIX shell script of randomized simulations.** Bash script used to perform simulations of gene family evolution. In .sh format, can be run from any UNIX terminal. Additional file 3:
**Appendix S3.** R script used within the shell script **(Appendix S2)** R script used within the bash script (Additional file 2). Can be open in R or any text editor.
