# Supplementary material for: Amino acid transporter expansions associated with the evolution of obligate endosymbiosis in sap-feeding insects (Hemiptera: sternorrhyncha)
Source: BMC Evol Biol. 2015 Mar 25;15:52. doi: 10.1186/s12862-015-0315-3 (PMC4374396; doi:10.1186/s12862-015-0315-3)
Supplement: Additional file 1: — Supplementary methods and displays. [file 12862_2015_315_MOESM1_ESM.pdf]

Additional file 1

Tree dating calibration and Bayesian priors

The nodes used to calibrate the ML phylogeny (fig. S1) are identified in fig. S2 and the parameters used in the calibration are specified in table S2 [1].

CAFE model fitting under alternative topology

Models of gene family evolution were fitted in CAFE using a phylogeny with a paraphyletic Paraneoptera, similar to the topology in [1]. The best models were selected using likelihood-ratio tests, fitting the null distributions to χ^2 distributions with degrees of freedom equal to the difference in number of parameters between the two models.

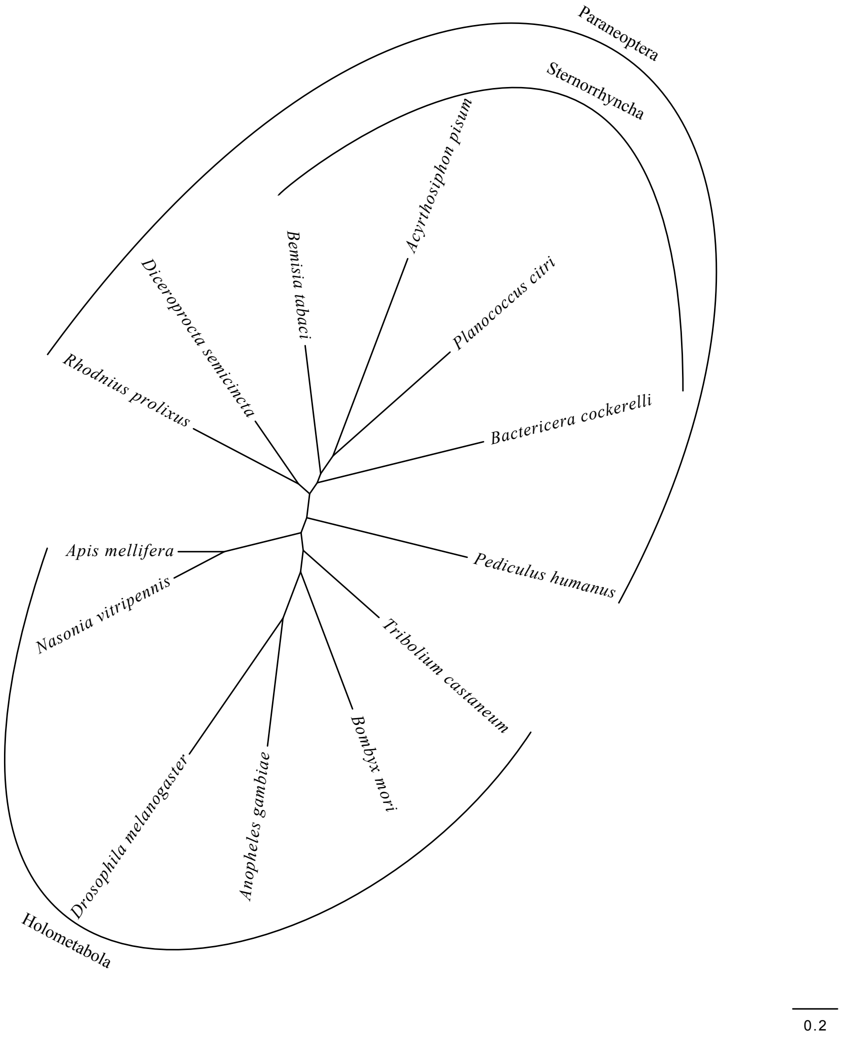

Figure S1: Unrooted ML phylogeny inferred in RAxML.

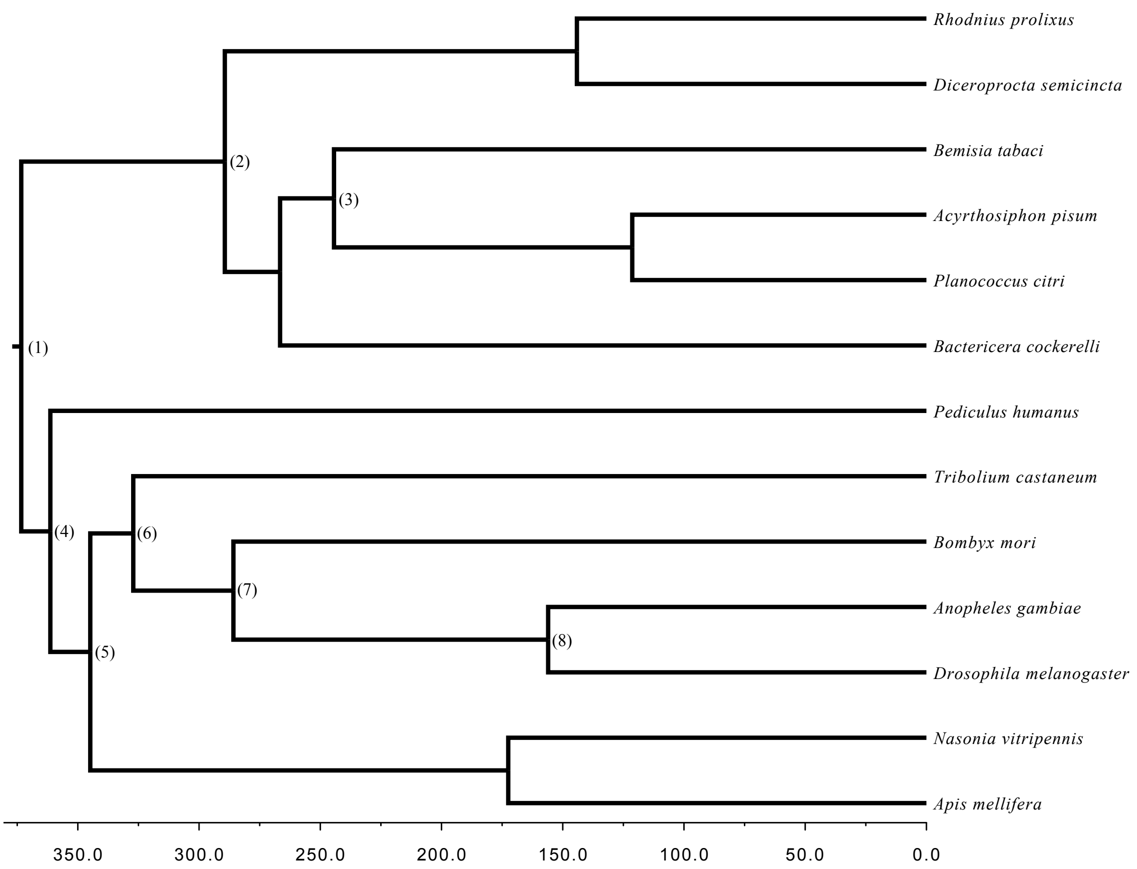

Fig. S2: Calibrated phylogeny used as starting point of Bayesian inference. The labeled nodes refer to the calibration point in table S2 used for the time calibration.

References

1. Misof B, Liu S, Meusemann K, Peters RS, Donath A, Mayer C, Frandsen PB, Ware J, Flouri T, Beutel RG, Niehuis O, Petersen M, Izquierdo-Carrasco F, Wappler T, Rust J, Aberer AJ, Aspöck U, Aspöck H, Bartel D, Blanke A, Berger S, Böhm A, Buckley TR, Calcott B, Chen J, Friedrich F, Fukui M, Fujita M, Greve C, Grobe P, et al.: **Phylogenomics resolves the timing and pattern of insect evolution**. *Science* 2014, **346**:763–768.

Table S1: Taxonomy of the species used in the phylogenetic inference.

<table>
<tr><th>Species</th><th>Superfamily</th><th>Sub-order</th><th>Order</th><th>Super order</th></tr>
<tr><td>Acyrthosiphon pisum</td><td>Aphidoidea</td><td rowspan="4">Sternorrhycha</td><td rowspan="6">Hemiptera</td><td rowspan="7">Paraneoptera</td></tr>
<tr><td>Planococcus citri</td><td>Coccoidea</td></tr>
<tr><td>Bemisia tabaci</td><td>Aleyrodidea</td></tr>
<tr><td>Bactericera cockerelli</td><td>Psylloidea</td></tr>
<tr><td>Rhodnius prolixus</td><td>Reduvidoidea</td><td>Heteroptera</td></tr>
<tr><td>Diceroprocta semicincta</td><td>Cicadoidea</td><td>Auchenorrhyncha</td></tr>
<tr><td>Pediculus humanus</td><td></td><td>Anoplura</td><td>Phthiraptera</td></tr>
<tr><td>Nasonia vitripennis</td><td>Chalcidoidea</td><td rowspan="2">Apocrita</td><td rowspan="2">Hymenoptera</td><td rowspan="6">Holometabola</td></tr>
<tr><td>Apis mellifera</td><td>Apoidea</td></tr>
<tr><td>Tribolium castaneum</td><td>Tenebrionoidea</td><td>Polyphaga</td><td>Coleoptera</td></tr>
<tr><td>Bombyx mori</td><td>Bombycoidea</td><td>Glossata</td><td>Lepidoptera</td></tr>
<tr><td>Drosophila melanogaster</td><td>Ephydroidea</td><td>Brachycera</td><td rowspan="2">Diptera</td></tr>
<tr><td>Anopheles gambiae</td><td>Culicoidea</td><td>Nematocera</td></tr>
</table>

Table S2: Nodes and divergence times used to calibrate the ML phylogeny. Node number refers to the labels in fig. S2. All dates in million years before present.

Node	Divergence lower bound	Divergence upper bound	Calibrated value
1	346	401	373.33
2	233	346	289.38
3	182	307	244.34
4	334	390	361.32
5	318	372	344.87
6	301	353	327.13
7	244	329	285.83
8	107	207	156.48

Table S3: Akaike Information Criterion (AIC) of models in the DupliPHY-ML algorithm.

	Model	# Parameters	ML	AIC
APC	Parsimony	1	24.13	50.26
	Parsimony+G	2	24.32	52.65
	BDO	2	23.88	51.76
	BDI	3	23.68	53.37
	BDI+G	4	23.88	55.76
	BDIE	4	23.68	55.36
	BDIE+G	5	23.88	57.75
AAAP	Parsimony	1	24.46	50.92
	Parsimony+G	2	24.66	53.31
	BDO	2	23.95	51.90
	BDI	3	23.75	53.50
	BDI+G	4	23.95	55.90
	BDIE	4	23.74	55.48
	BDIE+G	5	25.13	60.26

Table S4: Results of a Likelihood-ratio test comparing models with a global λ rate of gene duplication to models with a rate shift in Sternorrhyncha. The models were fitted using a phylogeny with a paraphyletic Paraneoptera (fig. S2). λ represents the probability of gene duplication/loss per gene per million years.

	Model	$\lambda_{Background}$ x 10^3	$\lambda_{Sternorrhyncha}$ x 10^3	Fold increase	-ML	# Parameters	Likelihood ratio	*p*-value
APC	Single λ	1.419	-	-	35.27	1	2.845	0.097
	Multiple λ	0.7235	3.4201	4.73	33.85	2		
AAAP	Single λ	1.3793	-	-	38.41	1	6.039	0.018
	Multiple λ	0.3774	3.7511	9.94	35.39	2		

Table S5: Results of the Likelihood-ratio test comparing models differentiating λ and μ to models with equal birth-death parameters. For APC, the estimated parameters are global across the phylogeny. For AAAP, the parameters were rates were allowed to shift in sternorrhynchan species. The models were fitted using a phylogeny with a paraphyletic Paraneoptera (fig. S2). λ represents the probability of gene duplication/loss (Single/Multiple λ), or gene duplication only (Single/Multiple λ + μ) per gene per million years. μ represents the probability of gene loss per gene per million years.

	Model	$\lambda_{Background}$ x 10^3	$\lambda_{Sternorrhyncha}$ x 10^3	$\mu_{Background}$ x 10^3	$\mu_{Sternorrhyncha}$ x 10^3	-ML	# parameters	Likelihood ratio	*p*-value
APC	Single λ	1.419	-	-	-	35.27	1	0.03	0.855
	Single λ + μ	1.3923	-	1.4517	-	35.25	2		
AAAP	Multiple λ	0.3774	3.7511	-	-	35.39	2	8.57	0.011
	Multiple λ + μ	1.14 x 10^{-10}	3.01	0.5096	1.8965	31.11	4		
